# 

**Published:** 1879-01

**Authors:** 


					﻿JANUARY, 1879.
TWENTY-SIXTH YEAR—No. 301.
HALL’S
Journal of Health.
PUBLISHED AT 141 EAST EIGHTH STREET,
(Near Bboadway),
JNTEXKT YORK.
E. H. GIBBS, A.M., M.D., Editor.
AGENTS FOB THE TRADE*.
AMERICAN NEWS COMPANY AND NEW YORK NEWS COMPANY
Terms, $1.50 a Year, or $1.00 for Eight Months. Postage paid.
SINGLE NUMBER, 15 CENTS.
Couch & Johnston, Printers, 11 Frankfort Street, New York.
				

